# Drug–Drug Interactions, Medication Adherence, and Stroke Should Be Considered When Approaching the Impact of Acid Suppression Therapy on Chronic Kidney Disease Patients. Comment on Chen et al. Impact of Acid Suppression Therapy on Renal and Survival Outcomes in Patients with Chronic Kidney Disease: A Taiwanese Nationwide Cohort Study. *J. Clin. Med.* 2022, *11*, 5612

**DOI:** 10.3390/jcm12010072

**Published:** 2022-12-22

**Authors:** Ai-Hsien Li, Yen-Ling Chiu

**Affiliations:** 1 Health Management Center, Far Eastern Memorial Hospital, New Taipei City 220, Taiwan; 2 Graduate Institute of Medicine, Yuan Ze University, Tao Yuan City 320315, Taiwan

**Keywords:** Histamine-2-receptor antagonist, proton pump inhibitor, chronic kidney disease

## Abstract

Chen et al. have published a report in this journal comparing the prognostic impact of a Histamine-2-receptor antagonist (H2RA) and a proton pump inhibitor (PPI) in patients with chronic renal disease. Based on Taiwan’s National Insurance Database, they concluded that those patients treated with the H2RA demonstrated a dose–response relationship of H2RA to reduced risk of ESRD and overall cardiovascular and non-cardiovascular mortality. In contrast, the CKD patients treated with the PPI were associated with an increased risk of overall mortality. However, from our point of view, there are some methodological and research concerns that need to be clarified by the authors. Otherwise, it would be too early to make a convincing conclusion.

Chen and colleagues have published in this journal recently a meticulously designed report to compare the prognostic impact of a Histamine-2-receptor antagonist (H2RA) and a proton pump inhibitor (PPI) in patients with chronic kidney disease (CKD) [1]. The authors made use of the Taiwanese national health insurance database to select patients with chronic kidney disease, dividing them into three groups based on the usage of the PPI or the H2RA during follow-up. They concluded that the CKD patients treated with the H2RA demonstrated a dose–response relationship of H2RA to reduced risk of ESRD and overall cardiovascular and non-cardiovascular mortality. In contrast, the CKD patients treated with the PPI were associated with an increased risk of overall mortality.

However, from our point of view, there are some methodological and research concerns that need to be clarified by the authors. Otherwise, it would be too early to make a convincing conclusion, as the authors have attempted to.

First, although Chen et al. already tried to correct the confounding effect of comorbidities by propensity score matching their study cohorts with coronary artery disease, hypertension, chronic liver disease, and acid peptic disease, they overlooked the potential roles of cerebrovascular disease and malignancy. Both factors have been proven to be important causes of death in CKD patients [2,3,4]. 

Second, PPI has been well known to have strong interaction with clopidogrel. Both PPI and clopidogrel compete for the CYp2C19 system, and the combination of these two drugs has been associated with hazardous effects on users [5,6,7]. Since clopidogrel has been an essential medication for patients with coronary artery disease, the combined prescription rate of clopidogrel in the study group should be addressed as well. 

Third, the prescription compliance of the acid-suppression medication, just as Chen and colleagues admitted in the context, was not assessed at all in this study. In another prospective observational study of 3305 adults with mild-to-moderate CKD who were enrolled in the Chronic Renal Insufficiency Cohort (CRIC) Study, the self-reported medication adherence rate was less than 70% [8]. 

Finally, the favorable outcome advantage of H2RA over PPI has been already reported in another patient group as well. In a meta-analysis enrolling 28,559 patients with critical illness, the PPI users have higher pooled relative risk of mortality than the H2RA users (1.05 (95% confidence interval 1.00–1.10)) [9].

With all these concerns addressed, we believe that this study will provide valuable evidence regarding the choice of PPI or H2RA in CKD patients.
